# Angiosarcoma in HIV-negative patients is not associated with HHV-8*

**DOI:** 10.1590/abd1806-4841.20164730

**Published:** 2016

**Authors:** João Avancini, José Antonio Sanches, Andre Pires Zanata Cherubim, Renato Pazzini, Cristina Mendes de Oliveira, Laura Masami Sumita, Neusa Yuriko Sakai Valente, Claudio Sergio Pannuti, Cyro Festa Neto

**Affiliations:** 1 Universidade de São Paulo (USP) – São Paulo (SP), Brazil

**Keywords:** Hemangiosarcoma, Herpesvirus 8, human, HIV

## Abstract

**BACKGROUND:**

Angiosarcoma is an aggressive, malignant neoplasm of vascular or lymphatic origin. Herpes virus 8 (HHV-8) is a member of the herpes family with a tropism for endothelial cells and it has been proven to induce vascular neoplasms, such as Kaposi's sarcoma. The role of HHV-8 in the pathogenesis of angiosarcoma has not been well defined.

**OBJECTIVE:**

To investigate the relationship between the presence of HHV-8 and angiosarcoma.

**METHODS:**

In this study, the team investigated the relationship between the presence of HHV-8, as determined by polymerase chain reaction, and angiosarcoma, using samples from patients with epidemic Kaposi's sarcoma as controls.

**RESULTS:**

While all control cases with epidemic Kaposi's sarcoma were positive for HHV-8, none of the angiosarcoma cases was.

**CONCLUSION:**

These findings support most previous studies that found no association between HHV-8 and angiosarcoma.

## INTRODUCTION

Angiosarcoma (AS) is an aggressive, malignant neoplasm of vascular or lymphatic origin. It is classified as a soft tissue sarcoma and accounts for 5.4% of all cutaneous sarcomas.^1^ AS is more common in the elderly, has no gender bias, and mainly affects Caucasians, with only 4% of cases involving black patients. ^1-3^

Typically, AS affects the head and neck, and most commonly the scalp.^1^ Despite originating from endothelial cells, it rarely affects the great vessels or the heart. Most AS lesions have a spontaneous origin, although a number of risk factors have been identified, including chronic lymphedema, radiation therapy, genetic syndromes and occupational exposure to chemicals like vinyl chloride.^1,4,5^ A notable increase in the number of AS cases over the last thirty years has emerged, which may be related to both an increased use of radiotherapy and improved diagnostic methods.^4,5^ The contribution of immunosuppression to this is uncertain, with only a few reported cases of AS in transplant or AIDS patients. ^5^

Herpes virus 8 (HHV-8), a member of the *Herpesviridae* family with a tropism for endothelial cells, is associated with vascular neoplasms in immunosuppressed patients, including those with epidemic Kaposi's sarcoma (EKS) or Castleman's disease, as well as the elderly (classic Kaposi's sarcoma). The role of HHV-8 in the etiology of AS remains unclear. ^1,6,7^ Thus, in this study, the team evaluated the association between HHV-8 and AS in Brazilian patients.

The aim of this study was to identify HHV-8 DNA in tumor samples from patients with AS and HIV-infected patients with EKS from a tertiary hospital in the city of São Paulo, Brazil.

## METHODS

The team retrospectively analyzed data from patients with AS or EKS whose diagnoses were based on clinical suspicion and confirmed through histopathological examination between 1992 and 2013. A total of 15 tissue samples from AS patients were selected. All AS patients were HIV-negative. Samples from 12 EKS patients were selected to match the AS samples in the same period. Histopathological samples from all patients were reviewed by an experienced dermatopathologist.

### DNA extraction

Four slices, each 10µm-thick, were cut from a formalin-fixed, paraffin-embedded tissue block and used for DNA extraction with the NucleoSpin Tissue Kit (Macherey-Nagel, Germany), following the manufacturer's instructions.

### Human β-globin polymerase chain reaction

To assess DNA quality and integrity, all samples were analyzed by polymerase chain reaction (PCR), using the PCO3+/ PCO4+ primers, to detect the presence of a 110 base pair (bp) fragment of the human β*-globin* gene. ^8^

### HHV-8 detection

Samples that were positive for human β*-globin* by PCR were further analyzed for the presence of four different HHV-8 genome regions. These consisted of two different fragments of the ORF-K1 variable-loop region, VR1 (380bp) and VR2 (336bp), and a 407bp fragment of the ORF-K12 region, using modified cycling conditions (initial denaturation of DNA at 95°C for 5 minutes; 40 cycles of 94°C for 50 seconds, 62°C for 50 seconds; and 72°C for 1 minute, followed by a final extension at 72°C for 10 minutes). ^8-10^ The fourth real-time PCR assay was designed to detect a fragment from the ORF-73 region.^11^

## RESULTS

Age, gender, ethnicity, and affected sites of the AS patients are summarized in table 1.

**Table 1:** Angiossarcoma patients: age, gender, ethnicity, and affected sites

Patient	Age (years)	Gender	Ethnicity	Affected site
1	85	female	Caucasian	scalp
2	72	male	Asian	head
3	68	female	Caucasian	head
4	69	female	Caucasian	arm
5	72	male	Caucasian	head
6	67	male	Caucasian	head
7	81	male	Caucasian	head
8	79	female	Caucasian	leg
9	66	male	Caucasian	scalp
10	70	female	Caucasian	head
11	70	female	Caucasian	leg
12	59	female	Caucasian	scalp
13	80	female	Caucasian	arm
14	75	male	Black	head
15	70	male	Caucasian	head

One of the 12 EKS samples and one of the 15 AS samples were excluded because no β*-globin* DNA could be detected (patients 14 and 25), indicating the absence of intact human DNA. Of the 11 samples from EKS patients tested for the presence of HHV-8 DNA, 10 entailed positive results. The patient sample that tested negative for HHV-8 DNA also had a very low level of β*-globin* DNA, which may explain why no viral DNA could be detected. The remaining 10 patients were used as positive controls.

In contrast to the samples from EKS patients, the 14 AS patient samples were all negative for HHV-8. These findings are summarized in table 2.

**Table 2:** HHV-8 analysis in patients presenting AS and EKS

Patient	Diagnosis	Histological review	β-globin	VR1	VR2	ORF-73	K12	HHV-8 result
1	AS	Confirmed	positive	-	-	-	-	negative
2	AS	Confirmed	positive	-	-	-	-	negative
3	AS	Confirmed	positive	-	-	-	-	negative
4	AS	Confirmed	positive	-	-	-	-	negative
5	AS	Confirmed	positive	-	-	-	-	negative
6	AS	Confirmed	positive	-	-	-	-	negative
7	AS	Confirmed	positive	-	-	-	-	negative
8	AS	Confirmed	positive	-	-	-	-	negative
9	AS	Confirmed	positive	-	-	-	-	negative
10	AS	Confirmed	positive	-	-	-	-	negative
11	AS	Confirmed	positive	-	-	-	-	negative
12	AS	Confirmed	positive	-	-	-	-	negative
13	AS	Confirmed	positive	-	-	-	-	negative
14	AS	Confirmed	negative	-	-	-	-	excluded
15	AS	Confirmed	positive	-	-	-	-	negative
16	EKS	Confirmed	positive	-	-	+	-	positive
17	EKS	Confirmed	positive	-	-	+	-	positive
18	EKS	Confirmed	positive	-	-	+	-	positive
19	EKS	Confirmed	positive	-	-	+	-	positive
20	EKS	Confirmed	positive	-	-	-	+	positive
21	EKS	Confirmed	positive	+	+	+	-	positive
22	EKS	Confirmed	positive	-	-	+	+	positive
23	EKS	Confirmed	positive	-	-	+	+	positive
24	EKS	Confirmed	positive	+	+	+	-	positive
25	EKS	Confirmed	negative	-	-	-	-	excluded
26	EKS	Confirmed	positive	-	-	+	-	positive
27	EKS	Confirmed	low	-	-	-	-	negative

AS, angiosarcoma; EKS, epidemic Kaposi´s sarcoma; HHV-8, herpes virus 8

## DISCUSSION

**Figure 1:**
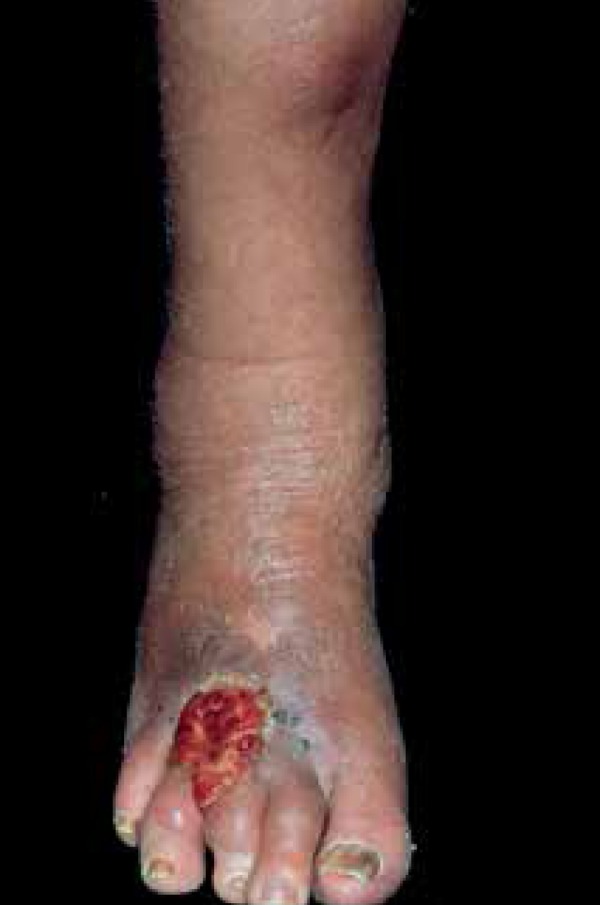
Angiosarcoma lesion in a patient with chronic lymphedema

The patient cohort in this study broadly matched the previously described epidemiological profile of this disease, with an approximately equal distribution between men and women (9 women and 6 men), a relatively advanced mean age (72 years) and a predominance of Caucasian patients (87% of the cohort was self-classified as belonging to this ethnic group).^1-3^ Likewise, with respect to tumor location, the majority of patients in this study (75%) had a primary AS in the head and neck region. The reason for this predilection is uncertain, but it is believed that ultraviolet rays may play a role.^12^ Four patients presented with AS lesions on the limbs, and the team identified chronic lymphedema in patients 8 and 11, a risk factor described for AS (Figure 1).^1^

A number of studies have investigated whether HHV-8 plays a role in the pathogenesis of AS, on the basis that this virus has a tropism for endothelial cells. HHV-8 was first isolated from patients with Kaposi's sarcoma (KS) and AIDS in 1994.^13^ It has oncogenic properties, but unlike other oncogenic viruses, it has a complex DNA-based genome, and infection not only leads to cell (endothelial) morphological changes, an increased growth rate, and extended life span, but it also causes the deregulation of angiogenesis, inflammation, and modulation of the immune system in favor of tumor growth.^14^

KS is a neoplasm with vascular proliferation that can present conventionally in immunosupressed patients or in patients who have previously undergone cancer treatment. It is endemic in some regions, but it can also occur epidemically due to HIV infection in immunosuppressed individuals. In all variants of KS, HHV-8 is implicated as the agent-inducing disease. The team therefore looked for a possible relationship between HHV-8 and other vascular neoplasms (such as AS), using EKS patient samples as positive controls for HHV-8 involvement.^13^

Soon after a relationship was discovered between HHV-8 and KS, McDonagh *et al.*^7^ published the first report on an association between AS and HHV-8 in 1996. Of the 24 cases selected from AS patients, 7 were positive for the presence of HHV-8 (29%), as were all the KS controls.^7^ This involved a series of cases, but subsequent positivity of HHV-8 in AS samples have only been reported in isolated cases, and there have been no further studies to corroborate this finding.^7,15-20^ Indeed, other studies have failed to find this association between HHV-8 and AS.^21-28^ A possible explanation for why only McDonagh *et al.* have found this association may be the higher prevalence of HHV-8 in Italy and Turkey, where the study was conducted.^29^

In 2005, Schmid and Zietz performed a study with 40 AS patients and also failed to find an association between HHV-8 and AS, although all the KS cases in this study were positive for the virus.^6^ Table 3 summarizes the previously published studies regarding the relationship between HHV8 and AS.^30^

**Table 3:** Previously published studies regarding the relationship between HHV-8 and AS

Authors	Number of cases	Positivity for HHV-8 in AS % (n)
Mc Donagh *et al.*^7^, 1996	24	29 (7/24)
Tomita *et al.*^21^, 1996	35	none
Dictor *et al.*^22^, 1996	10	none
Jin *et al.*^23^, 1996	15	none
Koizumi *et al.*^15^, 1996	2	50 (1/2)
Gyulai *et al.*^16^, 1996	1	100 (1/1)
Gyulai *et al.*^17^, 1997	1	100 (1/1)
Takata *et al.*^24^, 1997	10	none
Viviano *et al.*^25^, 1997	17	none
Lasota *et al.*^26^, 1999	33	none
Palacios *et al.*^27^, 1999	11	none
Karpati *et al.*^18^, 2000	1	100 (1/1)
Remick *et al.*^19^, 2000	1	100 (1/1)
Fink-Puches *et al.*^28^, 2002	19	none
Gessi *et al.*^20^, 2002	1	100 (1/1)
Kamiyama *et al*. ^30^, 2004	1	none
Schmid *et al*. ^6^, 2005	40	none
TOTAL	222	0.06 (13/222)

AS, angiosarcoma; HHV-8, herpes virus 8

In our study, none of the 14 AS cases was positive for HHV-8, in contrast to the EKS control cases, which were all positive for HHV-8. This is consistent with the findings of numerous other studies from different countries, which could not establish an association between HHV-8 and AS. Amongst the studies that did not identify HHV-8-positive AS, HIV serology was not addressed in 4 articles, which could make it more difficult to distinguish between AS and EKS. ^7,15,17,18^

The rate of HHV-8 infection varies worldwide, and the absence of HHV-8 in the AS lesions of the Brazilian patients described here reflects the findings of other studies in countries where the virus has a low prevalence.

## CONCLUSION

The Brazilian case series discussed in this report confirms the absence of HHV-8 in the AS lesions and adds data from a population not yet reported. Hence, despite the characteristic endothelial tropism of HHV-8 and its association with some vascular tumors, such as KS, it does not seem to be involved in the pathogenesis of AS.
